# Dr. Carson, on the Use of Digitalis Purpurea

**Published:** 1800-06

**Authors:** William Carson

**Affiliations:** Practitioner in Midwifery. Birmingham


### *To the Editors of the Medical and Phyſical Journal.*

GENTLEMEN,

I Have read with pleaſure your correſpondence on the various uſes of Digitalis Purpurea. I have exhibited it in Phthiſis Pulmonalis, and in ſeveral ſpecies of active Hæmorrhage, with unequivocal advantage.

Laſt October, I was called to Mrs. J——ns, aged 32, about three months gone in her pregnancy; by painful and reiterated coughing, ſhe expectorated much ſeeming purulent matter; her pulſe meaſured from a 100 to 110 ſtrokes in a minute: ſhe had likewiſe pains at intervals in the region of the uterus, attended with a profuſe diſcharge of blood. I preſcribed for her pills compoſed of opium, camphor, and digitalis. In three days, and by the time ſhe had taken five grains of digitalis, her pulſe was reduced to 70. The cough and expectoration now ceaſed to haraſs her; the uterine pain and hæmorrhage entirely ſubſided, nor did ſhe experience any relapſe. The ſecond of this month ſhe was delivered of a full-grown child.

The uterine affection in this caſe, may be conſidered as having been, in ſome meaſure, produced by the ſeverity of the cough and ſymptomatic fever; it was therefore only natural tural to hope, that by removing the caufes the effects would ceafe.

By reflecting on the above cafe, I have been induced to attempt the extenfion of fox-glove as a medicine; and in a line of practice where, fhould it prove fuccefsful, would add as many lives to the human fpecies, as phthifis pulmonalis deftroys, even adopting the much, by far too much, exaggerated proportion of Dr. Beddoes;—I mean, the prevention of abortion.

The increafe of the luxuries, and, perhaps, the increafe of the private vices of the prefent age, have given to the Britifh females an irritability of conftitution, which renders them liable to abortion from cafual, and often trivial, increafe of mental or corporeal exertion. In the female predifpofed to abortion, there is a peculiar irritability of the conftitution, and particularly of the uterus. The pulfe, in general, is quick, though feeble, and from caufes that would not affect the healthy female, quickened 20 or 30 ftrokes in a minute. The ufual medicines and regimen, directed as preventives of abortion, often fail; and in thofe females who have experienced two or three abortions, the profpect of future progeny becomes only a forlorn hope: thus, the peace and happinefs of families are impaired, and the lineal fucceffion of many noble and opulent families is deftroyed.

It would be difplaying a puerile enthufiafm, to hope much from the few imperfect trials which my practice, during the few laft months, have afforded me; and it would, no doubt, be confidered as arrogant, to pronounce a decifion on a fubject which, to eftablifh, would require the united practice of hundreds; yet, I cannot help indulging a flender hope, that, one day, the fox-glove will rank the firft medicine as a preventive of abortion.

By detailing the cafes and formula in which I prefcribed the digitalis purpurea, would only be needlefsly filling your ufeful pages; every medical man converfant in practice, and acquainted with medicine as a fcience, will know how to modify its dofe and formula to the exifting circumftances of his patient. I am,

GENTLEMEN,

Your very humble fervant,

WILLIAM CARSON, M. D.

Practitioner in Midwifery.

*Birmingham,*

*April* 12, 1800.

